# The association of education in a new society and social support from the education with the health of North Korean defectors: a cross-sectional study

**DOI:** 10.1186/s12889-021-10770-4

**Published:** 2021-04-21

**Authors:** Jihyun Lee, Yui Park, Dong-Hun Han, Ji Won Yoo, Wenlian Zhou, Pearl Kim, Jay J. Shen

**Affiliations:** 1 Department of Dental Education, School of Dentistry, Seoul National University, Seoul, 03080 South Korea; 2 Dental Research Institute, Seoul National University, Seoul, 03080 South Korea; 3 Department of Preventive and Social Dentistry, School of Dentistry, Seoul National University, Seoul, 08826 South Korea; 4 Department of Internal Medicine, School of Medicine, University of Nevada, Las Vegas, NV 89154 USA; 5 Department of Dental Medicine, School of Dental Medicine, University of Nevada, Las Vegas, NV 89106 USA; 6 Department of Health Care Administration and Policy, School of Public Health, University of Nevada, Las Vegas, NV 89154 USA

**Keywords:** Education, North Korean defectors, Self-rated health, Social support

## Abstract

**Background:**

The number of North Korean defectors (NKDs) escaping to South Korea has increased. The health status of NKDs is an essential factor for a successful settlement into South Korean society. However, no studies have been conducted on the health status of NKDs in terms of education and social support. The aim of this study was to determine the associations of education and social support with the self-rated health status among NKDs.

**Methods:**

This study utilized data gained from face-to-face interviews with 126 NKDs. A multivariable logistic regression and path analysis were performed to assess the effects of education in South Korea and social support on their self-rated health status and to explore the complex relationships between direct and indirect effects of the variables.

**Results:**

NKDs who did not experience regular education in South Korea responded that they were in poor health compared to their counterpart (OR = 5.78). Although a direct association between education in South Korea and self-rated health was not shown, there was an indirect path from education in South Korea to self-rated health through social support.

**Conclusions:**

Participation in regular education in South Korea is important for the health status of NKDs. Moreover, social support has an important role in the association between education and self-rated health. Social policies and NKD assistance programs should consider and reflect the combination of education and social support interventions relevant to the health status of NKDs.

## Background

North Korea’s economic difficulties after the fall and dissolution of communism in Eastern Europe and the Soviet Union and a great flood in 1995 that caused the Great Famine led to the deaths of millions of North Koreans due to starvation and hunger-related illnesses during the late 1990s [1]. Since then, many North Korean defectors (NKDs) have escaped to South Korea. The North Koreans’ defection to South Korea has been carried out through third countries such as Thailand and Laos via the North Korean-Chinese border, a relatively easy route compared to the direct route via North and South Korean border [2]. Despite the huge barriers and great danger, the number of NKDs entering South Korea has been steady at 1000 ~ 2000 annually since 2002, and the cumulative total by September 2019 was 33,247 [3].

Despite the financial and medical support by Korean Government, NKDs have experienced various difficulties in settling into South Korean society and managing their health conditions [4]. According to a survey of NKDs in 2018, 62.2% of NKDs suffered from stress in overall daily life, while 54.4% of South Koreans suffered from stress. Furthermore, 14.6% of NKDs had suicidal thoughts compared to 5.1% of South Koreans. Moreover, a particularly notable part was that the “physical/mental illness and disability” was the second highest cause of suicidal ideation (23.3%) [5].

Education can be considered as one of the most critical factors that potentially affect the adjustment of NKDs to South Korean society and their health. The health effects of education attainment against risk of morbidity and mortality are well established [6–8]. High education is shown to protect against a decline in self-rated health (SRH) [9, 10], chronic disease [11], and mortality [12]. As another essential component of human life, social support has been known to be associated with health and well-being. Social isolation entails a mortality risk comparable in magnitude to that of smoking, a sedentary lifestyle, obesity, and alcohol abuse [13].

Previous evidence suggests that education and social relationships interact with each other to affect health. Some researches in this area have found that those with high levels of education with more social support had better health outcome than their counterpart [14, 15]. Conversely, some studies found that the protective qualities of social support are stronger among individuals who are less educated [16, 17] and the other study suggested that social integration buffer the negative effects of low socio-economic status [18]. Moreover, the school dropout rate of NKD students was 3.0%, more than three times higher than the 0.94% for their counterparts (South Korean students). The report said that NKD students are having difficulty following their school classes because they cannot adapt to their school life [19]. Therefore, identifying the impact of the interaction between socio-economic status such as education level and social support on NKDs’ the health outcomes is a topic that should continue to be studied in the field of public health research.

Education is not only a determinant of health but also an opportunity to form social relationships. Especially for NKDs, being a part of a social community in South Korean society, such as a regular education in South Korea, provides them with good opportunities to receive social support and may have an important health-promoting impact. Previous studies [20, 21] showed that facilitating the visit of educational institutions for refugees is an essential factor for successful social inclusion. Although the health effects of education attainment against risk of morbidity and mortality are well established, the special situation of NKDs living in South Korea who use the same language but had lived in different social systems (North Korea) raises the need to analyze whether their educational experience in South Korea and social support are related to their health.

However, literature is scarce on the exact nature of the links among education in South Korea, social support, and self-rated health status among NKDs. We hypothesized that education in South Korea, social support, and self-rated health status would be directly associated with each other. We also hypothesized that education in South Korea would be directly and indirectly associated with self-rated health status through social support. Therefore, the aims of this study was to identify the effects of education and social support on the self-rated health status, respectively, and then to further assess the path from education in South Korea to self-rated health status through social support among NKDs.

## Methods

This was a cross-sectional study to assess the association of education and social support with self-rated health. It was approved by the Institutional Review Board of the Seoul National University School of Dentistry (No.S-D20170041). The written informed consent was obtained from all participants.

### Participants

The study participants were recruited with cooperation from the Hana Center, alternative schools for NKDs and the Catholic Bishop’s Conference of Korea National Reconciliation Committee in Seoul and Gyeonggi Province. There are 33,247 NKDs throughout South Korea and most of them are living in Seoul and Gyeonggi metropolitan areas. We thought that 40 NKDs from each level of education could be analyzed, so we planned to meet with 120 NKDs, and recruited more than 120 NKDs in consideration of those who refused to respond to the survey. A total of 137 participants were recruited from November 2017 to February 2018, and the purpose and method of this study were explained to them. The subjects were selected by a snowball sampling method which was used because North Korean refugees were often reluctant to reveal their identities. Therefore, recruiting refugees who had already formed close relationships with those who already decided to participate in this study was the most appropriate option. All the participants voluntarily responded to the survey (response rate 100%), agreed to join this study and gave their written informed consent and the survey was conducted at the alternative schools for NKDs or the Catholic centers where study participants could respond to the survey in a familiar and comfortable manner. The criteria for selection and exclusion were as follows.

- Selection criteria.
  - those who completed education at the Hana center.
  - those willing to participate in this study.
- Exclusion Criteria.
  - those who cannot understand and respond to the questionnaire by themselves due to cognitive problems.
  - those with a severe mental illness condition.
  - those who no longer want to participate in the research during the course of the study.

In the process of missing value handling, 11 cases were deleted and the final sample size for analyses were 126.

### Variables

The dependent variable was self-rated health status. It was measured with a 5-point scale (very good, good, moderate, poor, and very poor) and reclassified into two groups (good/moderate and poor).

Independent variables were education in South Korea and social support. Education in South Korea was reclassified into 1) college or more, 2) middle/high school, and 3) no education experience in South Korea. Social support score was measured by the Korean version of the Duke-UNC social support questionnaire which was developed by Suh et al. [22] The tool consists of 13 questions with a 5-point scale. After summing up, the score ranges from 13 to 65 points, and a higher score indicates more social support. The reliability of the Korean version of the Duke-UNC social support questionnaire was a Cronbach’s ⍺ = 0.89 at the time of its development. Social support was categorized as a lower/higher group based on the median score (34 point).

Confounding variables were age (continuous), gender (male/female), living with family (yes/no), residence period in South Korea (continuous), smoking (yes/no), and drinking (yes/no). Residence period in South Korea was calculated by months from entrance date into South Korea to October 2017 when the data collection was completed.

### Statistical analysis

After excluding missing values, 126 cases were included in the final sample for analysis. The characteristics of the participants were analyzed using frequency, percent, means, and standard deviations. Chi-square tests for categorical variables and independent t-tests for continuous variables were used to assess the associations of education in South Korea and confounders with subjective poor health (Table 1). The association between social support and self-rated health status (Table 2) and the association between social support and education in South Korea (Table 3) were evaluated by analysis of variance (ANOVA) and analysis of covariance (ANCOVA) controlling for confounders, respectively. Then we presented odds ratio (OR) and 95% confidence interval (CI) in Table 4. The association between education in South Korea and subjective poor health was assessed using logistic regression models adjusting for the above mentioned confounders (Model 1 in Table 4). Social support was regarded as a mediator, and logistic regression analysis was done controlling for confounders in model 1 and social support (Model 2 in Table 4). The role of the social support was evaluated by the percent (%) excess odd explained, which was calculated as [(OR_*adj confounders*_ – OR_*adj confounders + mediator*_) / (OR_*adj confounders*_ − 1) X 100 (%)] in this study [23]. The % excess odd explained represents the degree to which a mediator explains the relationship between education in South Korea and subjective poor health. Finally, to assess the direct and indirect associations of the education of NKDs in South Korea on their health, we set up a model that hypothesizes that education in South Korea affects their SRH directly and indirectly by their perceived social support. A path analysis was conducted using AMOS 23.0 (Chicago, IL, IBM SPSS Inc). Path coefficients were estimated by maximum likelihood estimation. Maximum Likelihood Bootstrapping was conducted to test the significance of the indirect effect with bootstrap samples of 2000 and 95% bias-corrected confidence intervals. Model fit was evaluated with a set of indices.

**Table 1:** Related factors of self-rated health status among North Korean defectors (*n* = 126)

Independent Variables	Self-rated health status		*p*-Value
	good/moderate	poor	
	(*n* = 98)	(*n* = 28)	
Age (years)	29.88 [14.71]	48.89 [16.98]	< 0.001
Gender			
Male	29 [29.6]	2 [7.1]	0.029
Female	69 [70.4]	26 [92.9]	
Living with family			
No	25 [25.5]	10 [35.7]	0.288
Yes	73 [74.5]	18 [64.3]	
Residence period in South Korea (months)	67.35 [53.50]	97.93 [52.20]	0.009
Smoking			
Yes	19 [19.4]	3 [10.7]	0.286
No	79 [80.6]	25 [89.3]	
Drinking			
Yes	66 [67.3]	14 [50.0]	0.093
No	32 [32.7]	14 [50.0]	
Education in South Korea			
No	16 [16.3]	16 [57.1]	< 0.001
Middle/high school	46 [46.9]	7 [25.0]	
College or more	36 [36.7]	5 [17.9]	

*Notes.* Means for continuous variables are reported with standard deviation in brackets. Frequencies for categorical variables are reported with column percent in brackets. *p*-values were obtained by independent t-test in continuous variables and chi-square test in categorical variables

**Table 2:** Unadjusted and adjusted social support score according to self-rated health status

Social support score	Self-rated health status					*p*-value
	Very good (*n* = 10)	Good (*n* = 39)	Moderate (*n* = 49)	Poor (*n* = 18)	Very poor (*n* = 10)	
Unadjusted						
Mean	27.60^a^	32.00^ab^	34.27^abc^	36.67^bc^	41.30^c^	0.001
[SD]	[9.66]	[8.15]	[6.76]	[8.27]	[7.51]	
Adjusted						
Mean	28.47^a^	32.06^ab^	34.13^bc^	37.20^cd^	39.86^d^	0.007
[SE]	[2.37]	[1.18]	[1.07]	[1.84]	[2.47]	

Unadjusted means are reported with standard deviation in brackets. Adjusted means are reported with standard error in brackets. *p*-values were obtained by ANOVA and ANCOVA controlling for age, gender, living with family, residence period in South Korea, smoking, and drinking. *SD* standard deviation, *SE* standard error

**Table 3:** Unadjusted and adjusted social support score according to education in South Korea

Social support score	Education in South Korea			*p*-value
	College or more (*n* = 41)	Middle/high school (*n* = 53)	No (*n* = 32)	
Unadjusted				
Mean	31.29^a^	33.77^ab^	37.59^b^	0.004
[SD]	[8.45]	[7.77]	[7.50]	
Adjusted				
Mean	31.90	33.05	38.01	0.020
[SE]	[1.27]	[1.14]	[1.63]	

Unadjusted means are reported with standard deviation in brackets. Adjusted means are reported with standard error in brackets. *p*-values were obtained by ANOVA and ANCOVA controlling for age, gender, living with family, residence period in South Korea, smoking, and drinking. *SD* standard deviation, *SE* standard error

**Table 4:** Association between education in SK and subjective poor health

Independent Variables	Model 1		Model 2	
	Odds Ratio	95% CI	Odds Ratio	95% CI
Education in SK				
College or more	1.00	[Reference]	1.00	[Reference]
Middle/high school	2.05	[0.47–8.95]	2.11	[0.46–9.67]
No	**5.78**	**[1.26–26.57]**	3.95	[0.78–19.93]
Age				
(years, continuous)	**1.05**	**[1.00–1.09]**	1.04	[1.00–1.09]
Gender				
(reference = male)	5.53	[0.56–54.47]	8.20	[0.70–96.59]
Living with family				
(reference = yes)	2.48	[0.84–8.51]	1.58	[0.46–5.39]
Residence period in SK				
(months, continuous)	1.00	[0.99–1.02]	1.01	[0.99–1.02]
Smoking				
(reference = no)	3.23	[0.40–26.03]	5.85	[0.57–60.34]
Drinking				
(reference = no)	1.25	[0.36–4.35]	1.05	[0.28–3.90]
Social support				
(score, continuous)			**1.10**	**[1.02–1.18]**

Model1 was adjusted for age, gender, living with family, residence period in SK, smoking, and drinking

Model2 was adjusted for age, gender, living with family, residence period in SK, smoking, drinking, and social support. *CI* confidence interval, *SK* South Korea

## Results

Descriptive results are listed in Table 1. Age ranges from 13 to 74 years old (mean age: 34.1 years old) and 41.3% were in their twenties. The NKDs were dominantly females (75.4%) and most of them lived with family (72.2%). The range of the residence period in South Korea was 1 to 213 months (mean length of residence in South Korea: 74.14 months), and two thirds of the participants lived in South Korea for 1 ~ 5 years (37.3% for 12 ~ 59 months and 31.7% for 60 ~ 119 months). Smokers and drinkers were 17.5 and 63.5%, respectively.

Additionally, 78.6% of the participants completed a regular educational curriculum in South Korea. The average score of social support was 33.9 with a minimum score of 13 and a maximum score of 51 points. Subjective health conditions showed that “good” and “moderate” were 38.9%, respectively, which surpassed “poor” (22.2%).

ANOVA and ANCOVA results are presented in Tables 2 and 3. The association between social support and self-rated health status is shown in Table 2. Social support scores increased as self-rated health status deteriorated. Individuals with at least a college degree reported a lower social support score (that is, a high social support status) than those who had less than a college degree (Table 3).

In an adjusted logistic regression analysis controlling for age, gender, living with family, residence period in South Korea, and smoking and drinking, those who did not experience a regular educational curriculum in South Korea showed 5.78 odds of self-reported poor health (Model 1 in Table 4, 95% CI 1.26–26.57). This association was attenuated to 3.95 in model 2 of Table 4 (95% CI 0.78–19.93). The % excess odd explained for no educational experience in South Korea was 38.23% when social support was included in model 2.

**Fig. 1:**
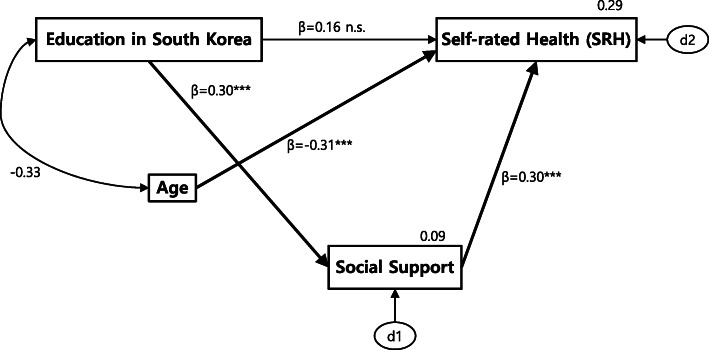
Path diagram of direct and indirect effects of Education in South Korea on Self-rated Health*.* ^*^p < 0.05, ^**^p < 0.01, ^***^p < 0.001

We evaluated the path model of direct and indirect effects between the three variables of interest, namely education in South Korea, social support, and self-rated health status along with a control variable age. As shown in Fig. 1 and Table 5, the path from Education in South Korea to SRH was not significant, in other words, the direct effect of education was not supported (*p* = 0.064). Paths from education to social support (β = 0.30, *p* < 0.001) and from social support to health (β = 0.30, *p* < 0.001) were all statistically significant, and the strength was big as the effect of age on health (β = − 0.31, *p* < 0.001). Thus, education in South Korea did not directly affect SRH but did indirectly affect it through social Support. The fit of the path model was assessed using the indices: χ^2^/df = 2.64; Normed Fit Index (NFI) = 0.945; Comparative Fit Index (CFI) = 0.961; and Standardized Root Mean Square Residual (SRMR) = 0.0107. All the indices were above the recommended criteria between very good and acceptable fit [24].

**Table 5:** Direct, indirect and total effects of Education in South Korea on Self-rated Health

Dependent Variable	Independent Variable	Direct Effect	Indirect Effect	Total Effect	*R*^*2*^
Self-rated Health	Education in South Korea	0.16	0.089^**^	0.25^**^	0.29
	Social Support	0.30^***^		0.30^**^	
	Age	−0.31^***^		−0.31^**^	
Social Support	Education in South Korea	0.30^***^		0.30^**^	0.09

^*^p < 0.05, ^**^p < 0.01, ^***^p < 0.001

## Discussion

This study analyzed the association of receiving education in South Korea and social support with self-reported health status among NKDs and the path among those three variables. This is the first study that observed the independent main effect of education received in South Korea on health among NKDs. Our findings indicate that receiving education in South Korea is positively associated with self-rated health status, even when potential confounding factors are taken into consideration. Like other populations, educational attainment is a significant predictor of morbidity, mortality, and other indices of physical health among NKDs [25–27]. Education in South Korea among NKDs is also significantly associated with social support. Those with college education in South Korea, relative to those without, had a higher level of social support. It is evident that education in South Korea is associated with psychological aspects of health that may be considered as an important part of social relationships.

Social support and networks are, in general, considered important protective factors relating to both mental and physical health [28, 29]. Immigrants or refugees’ social networks are often upheaved due to migration, and rebuilding social networks and ties may prove to be challenging. Our findings also indicate that social support is positively associated with better health. The buffering hypothesis suggests that social support affects health by attenuating the physiological effects of psychosocial stress [30]. Social support has been shown to have both buffering effects and direct beneficial effects on general morbidity [31, 32]. Evidence suggests that socioeconomic status (SES) and social relationships can interact with each other to affect health.

Finding of this study further supports that not only receiving education in South Korea is associated with the health status of NKDs—independence of socio-demographic factors and health behaviors—but that also social support has an important role in the association between education in South Korea and self-rated health among NKDs. However, we are not clear on the exact pathway from education to actual health which remains to be elucidated.

The hypothesis that is the most interesting to us is the potential effects of receiving education in South Korea on self-rated health either through social support or not. Our finding showed that education in South Korea did not directly affect their health, but education leads to social support, which affects their health. In other words, education affects health indirectly through social support. This result is not consistent with other studies [6–10] that argued a direct education effect on health. However, the significant path from education to social support and the one from social support to health were in agreement with previous studies. Evidence has supported that greater social support is associated with more education [33, 34]. Formal education may promote interpersonal skills and friendships and provide more resources (e.g., income and leisure time) to develop and maintain relationships [35]. Social support may also promote healthier lifestyles by enhancing individuals’ motivation, normative environment, and access to information [36–38]. The significant indirect effect of education on health through social support is in the same line with studies showing that education and social relationships interact with each other to affect health [15, 18]. It is noteworthy that the strength of this indirect effect of education (*β* = 0.25) and direct effect of social support (*β* = 0.30) on health is comparable to the strength of the age effect (*β* = − 0.31) on health. There could be many other mediating factors between education and health. In this study, we proposed social support as one of the factors. Further study is recommended to discover other factors that link education and health. Therefore, based on our findings, there is a need for a policy enforcing education and social integration activities in the NKD group for their health.

This study has limitations. First, the selection of study participants was restricted. Because the participants in this study were selected by a snowball sampling method, the study participants may not be considered as a representative sample of the NKD population. Moreover, the small sample size led to a wide range for the confidence interval in the results of the logistic regression analysis in this study. Therefore, the interpretation of the study results should be performed carefully. However, considering the limited opportunities to contact NKDs and restrictions on information collection from NKDs, these limitations are inevitable. Second, because this study was a cross-sectional survey research, it is difficult to infer causality among the variables. A well-designed longitudinal follow-up study will be needed to find underlying factors affecting the health status of NKDs on which proper health promotion and intervention strategies and programs can be developed. Third, it would have been better if the health outcomes were directly assessed by health professionals. However, the global measure of self-rated health is consistently identified as an independent and substantial predictor of actual health [39]. Nevertheless, it was still challenging for some NKDs with different social and cultural backgrounds to fully understand and answer the questionnaires. It is necessary to develop tools to better measure the health status of this vulnerable group.

## Conclusions

This study has some vital findings. The NKDs encounter challenges in adapting to a new political and social system in South Korea. To assist NKDs to overcome these challenges and improve their health condition, social integration policies and interventions focusing on enrollment in regular education curriculums/programs and participating in social interaction activities are essential. Further research should develop related intervention programs for NKDs and assess their outcomes.

## Data Availability

The datasets used and/or analysed during the current study are available from the corresponding author on reasonable request.

## Abbreviations

ANCOVA: Analysis of covariance
ANOVA: Analysis of variance
CFI: Comparative Fit Index
CI: Confidence interval
NFI: Normed Fit Index
NKDs: North Korean defectors
OR: Odds ratio
SES: Socioeconomic status
SRH: Self-rated health
SRMR: Standardized root mean square residual
